# Leicester Royal Infirmary

**Published:** 1919-09-13

**Authors:** 


### Leicester Royal Infirmary.

A series of new medical appointments is announced from the Leicester Royal Infirmary.

On the promotion of Dr. T. V. Crosby to be Honorary Physician, two vacancies offered for Assistant Physicians, and to these Dr. J. D. Slight, M.A., M.D., Edinburgh, and Dr. Arthur Foster, M.D., Edinburgh, Barrister-at-Law, have been appointed. During the war Dr. Slight has been in charge of medical patients, officers as well as other ranks, at the 5th Northern General Hospital, and has also acted as temporary physician to the Royal Infirmary. Dr. Foster has also been in charge of medical beds at the same military hospital.

For a vacancy as honorary assistant surgeon there were several applications, and the successful candidate proved to be Mr. R. S. Lawson, M.A., M.B., Ch.B., Edinburgh, F.R.C.S. England. Mr. Lawson graduated with first-class honours, and held a senior house surgeoncy at the Royal Infirmary, Edinburgh, and at the Royal Hospital for Sick Children, Edinburgh. He subsequently became medical superintendent and surgical registrar at the Dreadnought Hospital, Greenwich. He was one of the demonstrators and lecturers in anatomy at Edinburgh University, and acted as assistant lecturer and demonstrator in the Pathological Department there. He joined the Royal Navy in 1915, and after serving at sea for eighteen months was appointed operating surgeon at the Royal Naval Hospital, Chatham. At the end of eighteen months he went as operating surgeon in a hospital ship, and since demobilisation has been doing surgical work at Alder Hey Military Orthopædic Hospital.

